# Conditions potentially sensitive to a Personal Health Record (PHR) intervention, a systematic review

**DOI:** 10.1186/s12911-015-0159-1

**Published:** 2015-04-18

**Authors:** Morgan Price, Paule Bellwood, Nicole Kitson, Iryna Davies, Jens Weber, Francis Lau

**Affiliations:** Department of Family Practice, University of British Columbia, Vancouver, B.C. Canada; Health Information Science, University of Victoria, Victoria, B.C. Canada; Department of Computer Science, University of Victoria, Victoria, B.C. Canada

**Keywords:** Personal health records, Patient portals, Self-management, Systematic review, Chronic disease management

## Abstract

**Background:**

Personal Health Records (PHRs) are electronic health records controlled, shared or maintained by patients to support patient centered care. The potential for PHRs to transform health care is significant; however, PHRs do not always achieve their potential. One reason for this may be that not all health conditions are sensitive to the PHR as an intervention. The goal of this review was to discover which conditions were potentially sensitive to the PHR as an intervention, that is, what conditions have empirical evidence of benefit from PHR-enabled management.

**Methods:**

A systematic review of Medline and CINAHL was completed to find articles assessing PHR use and benefit from 2008 to 2014 in specific health conditions. Two researchers independently screened and coded articles. Health conditions with evidence of benefit from PHR use were identified from the included studies.

**Results:**

23 papers were included. Seven papers were RCTs. Ten health conditions were identified, seven of which had documented benefit associated with PHR use: asthma, diabetes, fertility, glaucoma, HIV, hyperlipidemia, and hypertension. Reported benefits were seen in terms of care quality, access, and productivity, although many benefits were measured by self-report through quasi-experimental studies. No study examined morbidity/mortality. No study reported harm from the PHR.

**Conclusion:**

There is a small body of condition specific evidence that has been published. Conditions with evidence of benefit when using PHRs tended to be chronic conditions with a feedback loop between monitoring in the PHR and direct behaviours that could be self-managed. These findings can point to other potentially PHR sensitive health conditions and guide PHR designers, implementers, and researchers. More research is needed to link PHR design, features, adoption and health outcomes to better understand how and if PHRs are making a difference to health outcomes.

## Background

### Personal health records

Personal Health Records (PHRs) are electronic health records controlled, shared, or maintained by patients to support patient centered care [1]. While PHRs have variable designs and features, they share a similar goal of improving patient engagement in their care. PHR enabled management can include both self-management and communication with members of the patients’ circles of care. PHRs can be standalone or tethered to another clinical information system such as a hospital information system or part of a regional electronic health record. PHR features can range from administrative (e.g. booking appointments and paying bills) to more clinical features (e.g. reviewing information, communicating with the care team, documenting care activities/results/outcomes). The potential for PHRs to reduce care costs and increase access to care is significant and it has been suggested that PHRs will help enable and empower patients [2,3]. However, despite millions of dollars spent on PHRs, the published evidence and research on PHRs is relatively limited [4,5], and, compared to the promises, adoption rates continue to be lower than hoped [6].

The evidence for benefit of PHRs is mixed [7]. There has been early positive evidence as well as dramatic challenges in adoption of PHRs [8]. Potential benefits of PHRs include improvement in: quality, access, and costs [9]. Stakeholders (e.g. patients, providers, payers) will experience benefits differently [10]. Several reviews have looked at aspects of PHRs and PHR features such as: benefit of secure messaging [11], medication adherence reminders [12], or symptom reporting [13]. Others examined effect on chronic diseases [7] or mental health [14]. The challenges to achieving PHR benefits include: poor adoption rates [6], poor integration into care processes [15], and policy limitations [16]. More work is needed to understand how PHRs can be meaningfully used [5] and how PHRs can support select patient populations with specific conditions.

The variable benefits seen with PHRs are due to a number of factors. The *PHR Adoption Model* describes four factors that can influence behavior which may lead to outcome changes: personal factors, technology factors, environmental factors, and chronic disease factors [6]. It highlights *chronic disease* factors as an important aspect of adoption of PHRs. That is, the nature of the chronic condition the patient has impacts adoption and value of the PHR.

For this paper we sought to discover which health conditions have been assessed for improvements in outcomes that correlate with PHR use. There has been a recent review of PHRs and chronic disease [5] but there has not been a review to examine *which* conditions have evidence of benefit from PHR use. A condition is an aspect of a person’s health including a symptom, illness, diagnosis, or health goal. Benefits could be considered for the person, the care team (both formal and informal), or the overall healthcare system.

### Objectives of this paper

The purpose of this paper is to add to our shared knowledge on PHRs by systematically reviewing the literature to develop an evidence-based list of conditions that have evidence of improvements that correlate with PHR use. We seek to answer the following questions:

1. *What health conditions have evidence for benefits of PHR enabled self-management?*
2. *What are the common care activities related to these conditions that are supported through the use of PHR?*
3. *Can we use these characteristics to predict other potentially PHR sensitive conditions?*

## Methods

### Evidence collection

Medline and CINAHL were searched for articles from 2008 to 2014. This focused findings on technically more modern PHRs (e.g. potential for mobile user experiences, more advanced web interactions). Search terms used: Personal Health Record or Patient Portal in the Title or Abstract. We limited our search to English language and abstract availability. Ethics was not required for this systematic review.

### Study selection and inclusion/exclusion criteria

Inclusion criteria were:

1. Use of terms personal health record, patient-controlled electronic health record, or patient portal in the title or abstract; *and*
2. Conditions or self-care activities; *and*
3. Evidence of actual use of PHR in specific conditions
4. Use of PHR in outpatient environment
5. Only primary studies were included that assessed benefit of PHRs by patients for those chronic conditions.

As we were seeking to find empirical evidence of PHR use and benefit, we excluded studies that did not have patients using PHRs (e.g. surveys on intention to use) or studies that were based only on usability testing. Further studies that assessed training effectiveness, or studies that only measured PHR use without looking at impact were excluded. We excluded any opinion, commentary, reviews, or theoretical PHR papers. Papers that evaluated electronic health records without focusing on PHR were also excluded.

Article selection occurred in two passes. First, the Titles/Abstracts were screened; the full text papers were pulled for those that passed initial screening for full review. Both screening and full text review were completed independently by two of the authors.

### Evidence synthesis

**Figure 1:**
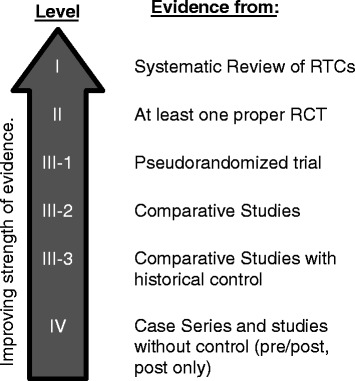
Extended NHMRC evidence hierarchy [17].

Two authors coded the included articles independently. The papers were graded using an extended evidence hierarchy based on Australia’s National Health and Medical Research Council (NHMRC) evidence hierarchy (Figure 1) [17]. Data was extracted from the papers including: type of PHR, patient population, health condition(s) examined, self-care activities, PHR features, and benefits observed (if any) as determined by the researcher. The codes were then compared. Consensus was reached on the coding for each characteristic. A third author was available for mediation if consensus could not be reached. The original authors, not the reviewers, determined benefits.

## Results

### Evidence of PHR benefit

**Figure 2:**
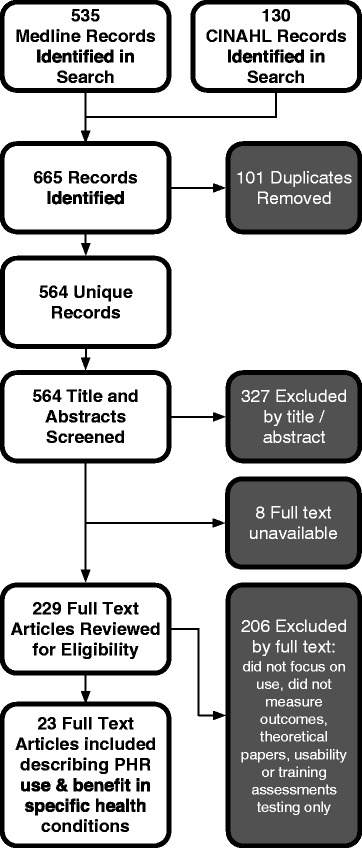
Literature Review Strategy, based on PRIMSA.

Our search followed PRISMA guidelines (Figure 2) found 564 unique records, of which 23 met inclusion criteria and examined a specific health condition. Two papers [18,19] each examined three specific conditions and were included. Within the 23 studies, there were seven randomized control trials, the rest were quasi-experimental or observational studies. Most studies were small and/or of short duration with no prospective study lasting more than one year. The metrics examined varied between studies such that comparison was difficult. 12 studies looked at self-reported data alone, with six studies using at least one previously validated instrument. Nine of the included studies looked at condition specific indicators such as A1c, LDL, plasma HIV-1 RNA, and blood pressure. These were tracked through chart reviews or electronic record reporting tools. The included studies are summarized in Table 1.

**Table 1:** **Summary of included primary PHR studies that measured benefit from use of PHR by patients**

**Author**	**Conditions**	**Benefit**	**Level**	**# of patients**	**Study design and duration**	**Location**	**PHR type and features**	**Evaluation methods**	**Reported Benefits**
Wiljer, 2010 [33]	Cancer	No	IV	320 consented, 114 completed study	6 weeks	Canada	Tethered PHR with access to personal health data (labs and diagnostic imaging), access to support groups and a virtual librarian.	State-Trait Anxiety Inventory; Stanford Self-Efficacy for Managing Chronic Disease	No change
Wade-Vuturo, 2013 [34]	Diabetes	Yes	IV	54 patients	Crossectional: PHR use >1 year in 43 patients	USA	Tethered Portal with secure messaging, access to medical records	Patient Self-Report; Chart review to assess glycemic control (A1c).	Improved Patient Satisfaction with Care
									Improved Disease Control
									More effective face-to-face visits
									Better Pt-Provider Communication
Urowitz, 2012 [21]	Diabetes	Yes	IV	17 patients	Crossectional, at least 6 months of access to PHR	Canada	Standalone PHR with ability to record personal health information and see trends, can also look up health information references.	Patient Self-Report	Improved Access to own information
									Improved access to pt information by provider
									Improved ability to self manage
									More Activated Patient
Tenforde, 2011 [35]	Diabetes	Yes	IV	10,746 adult patients	Retrospective audit over 12 months	USA	Tethered PHR with secure messaging and access to health record data, reminders for follow up and health information	Chart review for diabetes indicators (A1c, LDL-C, BP, BMI).	Improved Disease Control
Wald, 2010 [36]	Diabetes	Yes	II	2027 patients	prompt 3 weeks prior to encounter.	USA	Tethered PHR with secure messaging, access to health record data, Journal, and health information.	Patient and Provider Self Report	Improved Patient Satisfaction with Care
									Improved access to pt information by provider
									More effective face-to-face visits
									More Activated Patient
Hess, 2014 [37]	Diabetes (able to extract from paper)	Yes	IV	504 patients	Pre post, one year	USA	Tethered PHR with reminders for preventive care	Patient documentation of care received	Improved Disease Control
Fonda, 2009 [38]	Diabetes	Yes	II	104 patients	RCT, 52 weeks	USA	Tethered PHR with secure messaging, access to personal health data, educational materials.	Problem Areas in Diabetes (PAID) validated survey	Decreased Patient Distress
Lau, 2014 [39]	Diabetes	Yes	III-3	50 users and 107 non-users	6-24 months	Canada	Standalone PHR with health information, journaling, entering health data, secure messaging with providers	Chart review to monitor A1c control	Improved Disease Control
Sarkar, 2014 [40]	Diabetes	Yes	III-3	8705 users with 9055 matched reference group	Observational cohort study, 1 year	USA	Tethered PHR with access to record, secure messaging, renewal requests, and online scheduling.	Measured renewal rates for statins over 1 year based on chart data	Improved Disease Control
Wald, 2009 [41]	Diabetes	Yes	IV	37 patients	2 week follow up, patients were already using the general PHR as part of inclusion.	USA	Tethered PHR with secure messaging, access to personal health data, decision support, ability to annotate their health record, care plan.	Self Report	Improved access to pt information by provider
									More effective face-to-face visits
									Better Pt-Provider Communication
Grant, 2008 [20]	Diabetes	No	II	244 patients	RCT, use of PHR 52 weeks	USA	Tethered PHR with access to personal health data, decision support, care plans	DM indicators: BP control, A1c, LDL-C’ # of primary care visits.	No change
van Empel, 2011 [42]	Fertility	Yes	IV	369 couples	Cross sectional survey	Netherlands	Tethered PHR with secure messaging, access to personal health data, social support/forums.	Patient Self-Report, Partner Self-Report	Improved Continuity
									Improved access to health knowledge
									Better Pt-Provider Communication
Boland 2014 [43]	Glaucoma	Yes	II	38 intervention; 32 control	RCT; 3 months	USA	PHR that could record patient information and medications; daily reminders by text/phone to intervention group to take medication	Adherence monitoring with medication smart cap, patient surveys.	Improved medication management
Crouch, 2014 [44]	HIV	Yes	III-3	40 (20 users, 20 non-users)	Cross sectional	USA	Tethered PHR with access to labs, notes, secure communication and medication renewal.	Patient Activation Measure	More Activated Patient
									Improved Disease Control
Gordon, 2012 [45]	HIV	No	IV	112 active users	Survey, access up to 114 weeks	USA	Tethered PHR viewer with access to personal health data.	Patient-Self Report	Improved Access to own information
									Improved access to health knowledge
									More Activated Patient
Kahn, 2010 [46]	HIV	Yes	IV	221 users registered	cross sectional survey, access to PHR up to 21 months	USA	Tethered PHR with access to personal health data, ability to record own health data, access health information	Patient Self-Report	Better Pt-Provider Communication
									Improved ability to self manage
									More Activated Patient
McInnes, 2013 [47]	HIV	Yes	IV	1871 patients	Cross sectional survey and chart review	USA	Tethered PHR with access to personal health data, request medication renewal, reminders for preventive care, scheduling appointments, secure messaging	Chart review, survey data from Veterans Aging Cohort Study	Improved ability to self manage
Shade, 2014 [48]	HIV	Yes	IV	Unclear at site using PHR	12 month (6 pre and 6 post) study	USA	Standalone PHR with continuity of care patient summaries including HIV results; secure provider communication.	Chart review	Improved ability to self manage
									Improved Disease Control
Wagner, 2012 [49]	Hypertension	No	II	453 users	RCT, PHR use up to 39 weeks (4 visits)	USA	Tethered PHR with secure messaging, access to personal health data, track personal health data, access to health information, care plan goal setting.	Patient Self-Report, Chart review for blood pressure	No change
Chiche, 2012 [50]	Idiopathic thrombocytopenic purpura (ITP)	No	III-2	43 patients	26 weeks	France	Standalone PHR with ability to record personal health data	ITP patient assessment questionnaire	No change
Miller, 2011 [51]	Multiple Sclerosis	No	II	204 patients recruited	RCT, 52 weeks	USA	Standalone PHR with ability to record personal health data and receive decision support (through MS Quality of Life Inventory)	Sickness Impact Profile, MS Functional Composite, Control Subscale of the MS Self-Efficacy Scale	No change
								Seniors’ General Satisfaction and Physician Quality of Care	
								Euro-Quality of Life 5	
Solomon, 2012 [18]	Asthma, Hypertension, Diabetes	Yes	II	201 patients	12 week	USA	Tethered PHR with secure messaging and targeted health education weekly training modules.	Patient Activation Measure 13 (PAM-13)	Improved ability to self manage
								Chart Review	More Activated Patient
Sobko, 2011 [19]	Diabetes, hypertension, lipids	Yes	IV	9504	Cohort study - chart review 6 month pre and 14 months post PHR deployment	USA	Tethered PHR with access to health record, secure communication, decision support, medication renewal	Chart review: medication possession rates; A1c, blood pressure, lipids	Improved ability to self manage
									Improved Disease Control

### Health conditions evaluated

Ten health conditions were found in the included studies (Table 1). Seven of these ten health conditions had at least one study reporting benefit from the use of a PHR: **asthma, diabetes, fertility, glaucoma, HIV, hyperlipidemia, and hypertension**. Diabetes was the most studied condition with eleven of twelve studies showing benefit. Three conditions had studies that meth the criteria but did not show benefit of the PHR: cancer, idiopathic thrombocytopenic purpura (ITP), and multiple sclerosis.

### PHR supported care activities and PHR characteristics

74% (17/23) studies used tethered PHRs, connected to regional electronic medical records/electronic health records. 76% (13/17) studies that used tethered PHRs reported benefit. In comparison, only 50% (3/6) studies that used standalone PHRs showed benefit.

Studies described a set of PHR supported care activities that included the following:

- **Access Own Health Data** – Using the PHR to access shared clinical records. This could include view only (e.g. lab results) or editing/annotating.
- **Access Health Information** – Using the PHR to access handouts, protocol information or other self-management information in a linked or embedded knowledge base.
- **Record Personal Health Data** – Using the PHR to record and track subjective experience data or objective data related to the condition over time.
- **Receive Personal Decision Support –** Using the PHR health data to drive evidence-based reminders and alerts to the user to support self-management.
- **Plan Care** – Using the PHR to proactively set personal goals, targets and tasks related to health and care. For example: set weight or blood glucose targets.
- **Self-Manage Care** – Using the PHR to make day-to-day decisions about care management, such as medication dosing, food choice.
- **Communicate with Care Team** – Using the PHR to engage with and support members of the circle of care. This can be virtual and/or face-to-face. This includes direct communication (e.g. secure messaging) or sharing of data in a shared repository.
- **Communicate with Support Group** – Using the PHR platform to securely engage in communication with informal members of the care team or members of a community for support. Using a secure forum to discuss health related issues.

Table 2 highlights the types of PHR features that were reported by the conditions in studies where benefits were reported. Most reported using PHRs that provided patients with access to general health information (5/6 conditions) and improved communication with their provider(s) (4/6 conditions). Access to personal health data and ability to record or annotate against that data were supported for 3/6 conditions.

**Table 2:** **Summary of reported PHR features by condition**

**PHR Feature**	**Asthma**	**Diabetes**	**Fertility**	**Glaucoma**	**HIV**	**Hypertension**
Access Medical/Health Record		X			X	
Access Health Information	X	X	X		X	X
Record Personal Health Data		X		X	X	
Annotate Medical/Health Record		X				
Receive Personal Decision Support		X		X		
Develop/Manage Care plans		X		X		
Communicate with Provider	X	X	X			X
Communicate with Support Group			X			

No study described a PHR platform that included all eight features. Both positive and negative PHR studies described PHR platforms with various combinations of these features (see Table 1). It was not clear from many of the papers how these features were designed or implemented in the context of the healthcare system.

### Benefits and harm of PHR use

70% of studies (16/23) reported benefits associated with PHR use. Of the 16 studies that reported benefit, six were based only on self-reported data (or provider or partner reported) and not on objective data or a validated reporting tool. Of the six studies that relied on non-validated self-report, 83% reported benefit (5/6). By contrast, only 50% (5/10) of studies that used validated/objective data reported benefit. 57% (4/7) of Randomized Control Trials (RCTs) reported benefits although one of the RCTs used self-report data only.

The studies looked at a range of metrics that covered several domains of benefit from disease specific measures to validated surveys to custom surveys. Disease specific outcomes included primarily indicators for diabetes (A1c, LDL, blood pressure, and Body Mass Index) and one for blood pressure in a hypertension study.

One diabetes RCT [20] measured number of primary visits and saw no change with the PHR. Ten validated survey instruments were used across the 23 studies. Five validated survey instruments used were not specific to the health condition being assessed: the Patient Activation Measure, the State-Trait Anxiety Inventory, the Stanford Self-Efficacy for Managing Chronic Disease, Seniors’ General Satisfaction, Physician Quality of Care, and the Euro-Quality of Life 5. Five tools were health condition specific: Problem Areas in Diabetes (PAID) Survey (Fonda), ITP patient assessment questionnaire, Sickness Impact Profile, MS Functional Composite, and the Control Subscale of the MS Self-Efficacy Scale. Several studies used non-validated tools to gather targeted self-report data from their participants. Custom surveys examined a range of concepts, including: assessing the PHR components, patient satisfaction, improvements in self-management, access to care, access to information, and sense of control.

The benefits are summarized in Table 3, based on the descriptions by the original authors. The counts exceed the number of studies as studies often assessed and reported on multiple benefits. Most commonly reported benefits included more activated patients, improved ability to self-manage, and improved communication with providers.

**Table 3:** **Summary of reported benefits of PHR for each condition**

**Reported Benefit**	**Asthma**	**Diabetes**	**Fertility**	**Glaucoma**	**HIV**	**Hypertension**
Improved Patient Satisfaction with Care		2				
Improved Disease Control		5			1	
Decreased Patient Distress		1				
Improved Continuity			1			
Improved medication management				1	1	
Improved Access to own information		1				
Improved access to health knowledge			1			
Improved access to patient information by provider		3				
More effective face-to-face visits		3				
Better Patient-Provider Communication		2	1		1	
Improved ability to self manage	1	1			3	1
More Activated Patient	1	2			1	1

Cell numbers indicate number of studies that measured benefit in that area by health condition.

No study reported on harm from using the PHR. Providers in one study voiced concern that patients assumed the providers were monitoring the PHR constantly and patients may not report a change in health status as they may assume the provider is aware through the PHR [21].

## Discussion

The intention of this systematic review was to discover from the literature a set of health conditions that were potentially “sensitive” to a PHR as a health intervention. That is, which conditions had empirical evidence that associated PHR use with improved health outcomes. While we found 70% of the 23 included studies reported benefit, the literature base is still small, with most of the PHR research focusing on intention to use, usability, and use characteristics. Most of the included studies in this review that focused on outcomes were quasi-experimental and focused on shorter-term or self-reported benefits. No study examined morbidity or mortality. Thus, there is a gap in high quality primary PHR research that focuses on longer-term outcome measures. This is somewhat expected, as electronic PHRs are still a relatively new and are rapidly changing. Research is needed to better understand the features of the PHRs and how they are used so that benefits are seen. Additional research is also needed to explore unintended consequences of PHR. None of the included studies assessed potential harms and, as we know from other literature, there can be unintended consequences when using health information systems. This is consistent with Health Information Systems research in general [22,23] and speaks to a greater need in health informatics research. PHRs are socio-technical systems that can change many aspects of care processes as well as care outcomes. Multi-methods research is needed to understand PHR impact and capture some of these unintended consequences. Larger studies are needed that assess sustained benefits of PHRs and impact on morbidity, mortality, and cost, and use multiple and mixed methods to better understand the impact of PHRs as health information systems [24].

### Potentially PHR sensitive conditions

**Figure 3:**
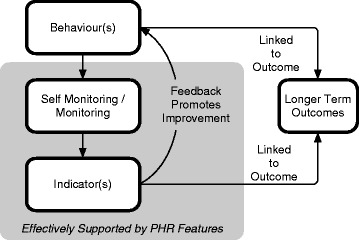
A model to describe how a PHR supports the monitoring of an indicator that promotes an effective behaviour change. If this loop is linked to a meaningful outcome and can be sustained, it can result in improvement outcomes.

From this review, there is early evidence that highlights a small group of conditions that have evidence of benefit to using a PHR as a health intervention. These conditions include: *diabetes, hypertension, asthma, HIV, fertility management, glaucoma, and hyperlipidemia*. Benefits were seen in care quality, access, and/or productivity. These conditions share several common characteristics: Each of these conditions is *chronic*. They have a significant benefit from self-management through *behavioural changes*. Many have an aspect of monitoring, either from the clinician or the patient (self-monitoring). *Self-management* is present in all. The seven conditions were conditions where the self-management behaviours could be suitably tracked in a PHR and were tightly linked to the feedback of monitoring/self-monitoring of indicators (Figure 3). For example, self-monitoring blood pressure in hypertension or glucose levels in diabetes allowed for more specific and direct feedback to patients using a PHR.

Given the early state of the evidence for PHRs, it is not possible to exclude other conditions from this list and, indeed, many of the other conditions that have been evaluated but did not show benefit (e.g. Cancer) have several similar traits to the other conditions that have supporting evidence. It is expected that the PHR design, implementation of the PHR in the context of those patients or the design of the study had an impact on discovering benefits. That is, if the patient or the health condition was not as well supported by the particular PHR or the implementation was different, benefits may not have been seen. Further, other health conditions could benefit from PHRs that have not been examined in studies included in this review. For example, obesity was not found in this this review but has significant prevalence [25,26] and results in elevated risk for specific conditions and morbidity [27,28]. Measurements such as body size measurements (e.g. waist size) and fat mass measurements can be used to monitor the impact of behavioural changes on obesity. It could benefit from PHR support. Indeed, there are several self-management applications that are providing tools for weight management such as: SapoFit [29] and over 200 smart phone apps [30].

### Contribution to knowledge

This work expands on Logue’s PHR adoption model, providing additional information on the *chronic disease factors* that influence PHR adoption [6]. The PHR activities list (Table 2) can serve as a model that can be mapped back to the management of other chronic conditions to help in the design and use of PHRs in the future. PHRs today have a range of features/value propositions [10].

For PHR designers and implementers, this condition list can serve as optional target populations (Figure 3) and can be used when considering PHR features. Designers can consider specific feedback loops for health conditions and consider how specific PHRs support PHR enabled care activities such as accessing health information, communicating with providers, and accessing and recording personal health information. Implementers can use the same model when considering how to implement a PHR within a healthcare system.

### Study limitations

Electronic PHRs are new and thus the evidence base is also new, with few studies and no large, long-term studies. Also, PHRs are different and changing, and new features, such as mobile devices and various environmental and personal sensors, are rapidly evolving. These features may well change the value propositions of PHRs. This review may have missed some studies that were not found through its search strategy. For example, there have been PHR related papers published prior to 2008 that were not included. Earlier reviews suggested that PHRs were limited in functionality [31] and we chose to focus on newer studies that may examine more robust PHRs that leverage Internet and mobile technologies. Quality of studies was graded but papers were not excluded based on quality. The studies focused on different aspects of benefit, limiting between-study meta-analysis. Application of a common evaluation framework in future primary studies would help build a common knowledgebase related to PHRs. PHR features and usability of the PHR were not always clear from the published studies, but we know that this will effect the realization of benefits [32]. It is expected that the variability in PHR design of features changes value [10]. Much of this information was not available in the studies. Finally, as a review, the list of conditions is limited to what has been studied and included in the search.

## Conclusion

While many factors can influence the impact of a PHR such as the PHR’s function and design, how it is implemented by the patient and by the healthcare system, we discovered some early evidence of benefit for seven health conditions: asthma, diabetes, fertility, glaucoma, HIV, hyperlipidemia, and hypertension. Each of these conditions are chronic in nature and tend to have clear feedback loops of behaviours resulting in changes in indicators that can be better self-monitored through the PHR. However, the current body of evidence for PHRs is small, with studies limited to assessing perceptions of benefit or early indicators. There is a need to continue research into how PHRs are designed, what features they have, how they are adopted as well as studies that assess PHR impact on health outcomes. Longer term and more robust studies are needed, and our current knowledge can guide future research to potentially PHR sensitive conditions.
